# Associations between smoking habits and weight-related behaviors among people who smoke in the United Arab Emirates: a cross-sectional study

**DOI:** 10.3389/fpubh.2026.1732881

**Published:** 2026-04-28

**Authors:** Sharifa AlBlooshi, Maryam Hassan Dawood, Dana N. Abdelrahim, Marika Lo Monaco, Falak Zeb

**Affiliations:** 1 Department of Health Sciences, College of Natural and Health Sciences, Zayed University, Dubai, United Arab Emirates; 2 Research Institute for Medical and Health Sciences, University of Sharjah, Sharjah, United Arab Emirates; 3 Department of Health Promotion Sciences, Maternal and Infant Care, Internal Medicine and Medical Specialties (PROMISE), University of Palermo, Palermo, Italy

**Keywords:** health behaviors, healthy lifestyle choices, nursing, public health, smoking cessation, smoking habits, weight management

## Abstract

**Background:**

Smoking is one of the leading global public health problems, and is complicated by multiple reasons for its prevalence and associated health problems. An understanding of the influence of smoking activity on weight management is essential to formulate successful health programs.

**Aim:**

The objective of the present study was the investigation of the association between smoking habits and weight management among adult people who smoke in the UAE.

**Methods:**

A cross-sectional study was conducted with 232 participants who completed a 36-item questionnaire assessing their smoking habits and weight management practices.

**Results:**

The sample consisted mostly of males (75.4%) aged 21–40 years, with the majority from Dubai (58.6%). There were statistically significant relationships between people who smoke, smoking duration, and frequency of weight management (*p* = 0.023), tobacco use and self-reported weight changes (*p* = 0.029), and smoking duration and BMI categories (*p* = 0.043). Conversely, shorter smoking exposure periods (<1 year) were associated with more variable BMI tracking and weight-based surveillance, as well as a higher likelihood of maintaining a normal BMI. Those who smoked weekly were more likely to report both perceived weight change and no weight change.

**Conclusions:**

These findings indicated that smoking prevalence is associated with weight-control behavior and BMI, especially among beginning people who smoke. In addition, smoking-cessation interventions must take a proactive approach to address weight concerns and self-monitoring behaviors earlier in the smoking process.

## 1 Introduction

Smoking is a global public health problem that has a substantial effect on personal health, resulting in a continuum of morbidities and mortality risks (1, 2). Some recent estimates of the prevalence of Emirati men and women smoking showed 24.3 and 0.8% in men and women, respectively (2). Importantly, the highest frequency was observed among men aged 20–39 years in the United Arab Emirates (UAE) (2). This suggests that smoking is a substantial health problem, even though rates are relatively lower compared with global averages (3). Smoking has detrimental effects not only on people who smoke themselves but also on people who do not smoke through exposure to second-hand smoke, thereby increasing the public health burden and a significant health risk (4, 5). Smoking prevalence is influenced by many factors, including intrapersonal, interpersonal, social, and environmental factors (6). Smoking-related behaviors are substantially influenced by intrapersonal (e.g., age, sex, personal attitudes to smoking) and interpersonal factors (e.g., family status, peer opinion) (7). Psychological variables, such as stress and anxiety, may also play a role in smoking urges (8). Additionally, smoking prevalence is strongly associated with community and organizational level factors such as policies and socio-economic conditions (6). Consequently, grasping these things is essential for creating efficacious solutions to lessen the growing incidence of smoking. The adverse health effects associated with smoking are well-documented, including diabetes, cardiovascular disease, and many other diseases. This is largely in response to carcinogenic tobacco components, well-established cancer stimulators (2, 4, 5, 9–11).

Previous studies examining the relationship between smoking behavior and weight management were inconclusive. Not all studies demonstrate a significant association between smoking behavior and weight control (12–14), and no clear link has been found to elucidate the substantial weight differences between cigarette-smoking individuals belonging to various smoking groups. Other studies also showed a marked inverse relationship between smoking and BMI, attributed to appetite suppression and increased energy expenditure (15–25). Moreover, several studies demonstrated a positive correlation between quitting smoking and weight gain (3). Individual differences in smoking behavior may facilitate weight control, but the overall causal relationship between smoking and weight remains complex and should be investigated (15, 26–39).

This research aims to investigate the link between smoking and weight control in the UAE among people who smoke. Obesity and overweight are significant public health issues in the UAE, and the data show that more than two-thirds of the population is affected (2, 30). Being overweight is most common in middle-aged people and men, and it is a risk factor for cardiometabolic diseases such as diabetes and cardiovascular diseases (2, 30). The rapid growth of urbanization, a high-energy diet, and low levels of physical activity have all contributed to this problem, which is why it is important to investigate weight-related behaviors in this setting (30).

Tobacco use in the UAE is practiced in a diverse product category that includes cigarettes, midwakh (dokha), shisha (waterpipe), electronic cigarettes (ECs), and heated tobacco products (HTPs) (1, 2). Although traditional cigarettes are still widely used, there is evidence of increasing experimentation with ECs in the region, especially among young adults and men, who are attracted to them because of their perceived lower risks and potential weight loss benefits (1, 2, 30). However, use of different tobacco products varies across groups, and there is no longitudinal data on trends in these products in the UAE (30).

This research is guided by the theory of planned behavior. The theory of planned behavior suggests that health behaviors are mainly influenced by behavioral intentions. Behavioral intentions, in turn, are influenced by attitudes, subjective norms, and perceived behavioral control. In this research, the theory of planned behavior has been used as an interpretive framework. In this research, smoking frequency and smoking duration have been used as behavioral indicators. On the other hand, weight monitoring practices, perceived weight change, and smoking cessation attempts have been used as proxy indicators. The term behavioral orientation refers to the general tendency of an individual toward self-regulation and health decision-making. The proposed relationship has been depicted by using a conceptual model.

The aim of this study is to quantify the relationship between smoking and weight-related behavior using smoking cessation, cultural factors, and lifestyle. The purpose is intended to look at this kind of crossroads of the community, as far as to identify patterns that could offer clues to the creation of interventions that are targeted and based on specific evidence that enhance the outcomes of quitting and weight problems of addiction-disordered populations. The key research question is: does smoking habit and weight management have any relation, and how does smoking cessation, culture, and lifestyle influence this relation? We hypothesize that smoking behavior is closely related to weight management behavior among adult people who smoke in the UAE; on the other hand, smoking cessation, cultural perceptions, and lifestyle shape the potential association between smoking and weight outcome in adult people who smoke.

## 2 Materials and methods

### 2.1 Ethical approval

Ethical approval was obtained from the Zayed University Ethics Committee (Code# ZU24_070_S). Informed consent was obtained electronically from all participants prior to their inclusion. Rigorous protocols were implemented for data handling, all data were securely stored in password-protected files, and sensitive information was permanently erased after analysis. Access to personal data was restricted, and stringent measures were in place to safeguard all storage devices against unauthorized access. Participants were assured of confidentiality and privacy of their personal information.

### 2.2 Study design

A cross-sectional design was used to collect data from February 2024 to April 2024 via an online survey with a closed-ended, self-reported questionnaire adapted from a tool used in a recent study conducted in Saudi Arabia (26). This tool demonstrated content and face validity through its structured design and successful use among 744 participants in a similar Middle Eastern context. Clarity and relevance of the survey were further confirmed through a pilot test involving 20 participants, with no changes required. The link to the online questionnaire was distributed through various social media platforms, including WhatsApp, Snapchat, and Instagram. Only current participants who smoke, are aged 18 or older, and live in the United Arab Emirates were allowed to participate, as per the survey invitation. Respondents were asked to affirm their current smoking status in the first survey item, which acted as a screening question. Those who replied that they did not smoke were immediately disqualified from continuing. A snowball sampling approach was employed to gather data from participants. We excluded participants who were under 18, non-smokers, and those who didn't complete the survey. The online survey was distributed to 300 people who smoke in the UAE, and participants were encouraged to share it with friends and family who were willing to participate. *A priori* sample size calculations for estimating proportions with a 5% margin of error at 95% confidence suggest a minimum sample of 384. Although the calculated sample size was not reached, a total of 232 participants were recruited during the study period. As the topic is sensitive, recruiting participants was challenging, as some individuals who smoke were reluctant to take part despite assurances of confidentiality and privacy outlined in the consent form.

Weight management was assessed using self-reported questionnaire items. Participants were asked whether they currently engaged in practices to control or manage their body weight and whether they perceived changes in their body weight in relation to smoking behavior. Specifically, participants were asked: “Do you currently engage in any practices to control or manage your body weight (e.g., dieting, physical activity, or other methods)?” (yes/no), and “Since smoking or attempting to quit smoking, how would you describe changes in your body weight?” (no change, weight gain, or weight loss). These items were used to characterize weight management behaviors and perceived weight changes among people who smoke.

The level of smoking behavior was measured by using self-report questionnaire items. The participants were asked about the frequency and duration of their smoking habits. They were also asked about the type of tobacco product they used. The frequency of smoking was measured by asking participants how often they smoke. The responses were recorded using categories. The participants were also asked about the type of tobacco product they used. The tobacco product used by the participants included cigarettes, midwakh or dokha, shisha or waterpipe, electronic cigarettes, and heated tobacco. The participants were allowed to choose more than one type of tobacco product. These are the questions with choices: tobacco use frequency: (daily, weekly, monthly). Daily tobacco intake: (less than five cigarettes per day, 5–10 cigarettes per day, more than 10 cigarettes per day). Smoking duration: (less than a year, 1–5, 6–10 years, more than 10 years) and tobacco product preference: cigars/cigarettes, E-cigarettes/IQOS, Midwakh, pipes/shisha.

### 2.3 Data collection and measurements

The survey comprised three sections contained 36 closed-ended questions in that: (i) self-reported anthropometric data related to body weight, height, BMI, physical activity and body perception; (ii) smoking habits, including tobacco use frequency, daily tobacco intake, smoking duration, and smoking cessation attempts; (iii) questions about sociodemographic data including sex, age, residency, nationality, marital status, education, and occupation. The question, “How often do you monitor or actively manage your body weight?” was used to assess weight management. The possible answers were daily, weekly, monthly, or less, and never. “How often do you currently use tobacco products?” (daily, weekly, or monthly) was used to assess smoking frequency, and “On average, how many cigarettes or tobacco equivalents do you use per day?” was used to gauge daily tobacco intake. To ensure that typical types of tobacco use in the UAE setting were recorded, participants were asked to indicate their major tobacco product preference, including pipes/shisha, midwakh (dokha), e-cigarettes/HTPs (e.g., IQOS), and cigarettes/cigars. Height and weight measurements were self-reported by participants and may be subject to reporting bias. The clarity of the questions was pre-tested in a pilot study with 20 non-randomly chosen participants. Responses were included in the final dataset because no modifications to the questions were made, and the inclusion criteria were met for the sample.

### 2.4 Data analysis

Statistical analysis for the present study was performed using the Statistical Package for the Social Sciences (SPSS, version 29; IBM Corp., Armonk, NY, USA). Descriptive statistics were used to report on participant characteristics, smoking patterns, eating behaviors, and health status. Continuous variables were expressed as means with standard deviations, and categorical variables as frequencies with percentages. Categorical variables were analyzed using their original response categories, as defined in the questionnaire. Categorical predictors were smoking duration (<1, 1–5, 6–10, >10 years) and frequency of smoking (daily, weekly, monthly). BMI was derived from self-reported weight and height and categorized according to WHO cut-offs. Associations between categorical variables were also assessed bivariately using the Chi-square. Regression analyses were performed to examine associations between smoking behaviors and weight outcomes across subgroups. To investigate the relationship between smoking time and the study's weight management frequency (daily, infrequent, or never), multinomial logistic regression was used. Binary logistic regression was applied to explore the relationship between the frequency of smoking and perceived weight change (change vs. no change) and between the duration of smoking and the BMI (kg/m^2^) groups (normal weight vs. overweight/obese). Odds ratios (ORs) and their associated 95% confidence intervals (CIs) were presented. A two-tailed *p*-value <0.05 level was considered to be statistically significant.

## 3 Results

This study involved 232 participants. As indicated in Table 1, the majority of participants were male (75.4%). The highest proportion of participants was adults, accounting for 33.2, 25.9, and 20.7% in the 21–30, 31–40, and 41–50 age brackets, respectively. Over half of the participants reside in Dubai (58.6%), followed by Abu Dhabi and Sharjah (13.4% each). Emirati nationals constituted 76.7% of the sample. Regarding educational background, 52.2% had a high school education or less, and 40.9% held a bachelor's degree. The majority (71.1%) were employed. Anthropometric data indicated a mean height of 1.71 ± 0.1 m and a mean weight of 75.76 ± 18.14 kg. The mean BMI was 26.03 ± 5.55 kg/m^2^. The body mass index was categorized according to the WHO guidelines as underweight (4.3%), normal weight (42.7%), overweight (37.5%), and obese (15.5%).

**Table 1:** Demographic information of the participants (*n* = 232).

Sociodemographic variables	Category	Frequency	Percentage (%)
Sex	Male	175	75.4
	Female	57	24.6
Age	18–20	26	11.2
	21–30	77	33.2
	31–40	60	25.9
	41–50	48	20.7
	>50	21	9.1
Emirates	Abu Dhabi	31	13.4
	Dubai	136	58.6
	Sharjah	31	13.4
	Others	34	14.7
Nationality	Emirati	178	76.7
	Non-Emirati	54	23.3
Marital status	Single	103	44.4
	Married	129	55.6
Educational level	High school or less	121	52.2
	Bachelor's degree	95	40.9
	Postgraduate (Master's or PhD)	16	6.9
Occupational status	Employed	165	71.1
	Unemployed	67	28.8
Anthropometric measurements	Mean ±SD	Min	Max
Height (m)	1.71 ± 0.1	1.43	2.00
Weight (kg)	75.76 ± 18.14	39.0	152.0
BMI calculated (kg/m^2^)	26.03 ± 5.55	14.33	49.63
BMI categories	Category	Frequency	Percentage (%)
	Underweight	10	4.3
	Normal	99	42.7
	Overweight	87	37.5
	Obese	36	15.5

SD, standard deviation; BMI, body mass index (kg/m^2^). BMI categories are based on WHO classification; Underweight: <18.5; Normal: 18.5–24.9; Overweight: 25.0–29.9; Obese: ≥30.0.

Table 2 presents the participants' smoking habits and weight management. Participants were classified by reported tobacco use, including single- and multiple-product use; however, analyses were not stratified by product-use combinations due to sample size constraints. It shows that 74.6% of participants used tobacco products daily. Additionally, 40.1% of people who smoke utilized tobacco less than five times per day, and nearly half of the participants (49.6%) reported smoking for more than 10 years. Additionally, the data illustrate that the most preferred tobacco products among participants are electronic cigarettes and E-cigarettes / IQOS (37.1%), followed closely by cigars/cigarettes at 35.3%. Regarding weight management, 43.1% of participants reported never monitoring or managing their weight. The highest percentage of the participants, at 40.9%, reported no changes in weight due to their smoking habit, while 37.1% indicated uncertainty about their weight. The highest percentage of participants was satisfied with their weight (36.6%), while 31.5% were dissatisfied.

**Table 2:** The smoking habits and weight management of the participants (*n* = 232).

Smoking habits	Category	Frequency	Percentage (%)
Tobacco use frequency	Daily	173	74.6
	Weekly	31	13.4
	Monthly	28	12.1
Daily tobacco intake	Less than five cigarettes per day	93	40.1
	5–10 cigarettes per day	69	29.7
	More than 10 cigarettes per day	70	30.2
Smoking duration	Less than a year	36	15.5
	1–5 years	51	22.0
	6–10 years	30	12.9
	More than 10 years	115	49.6
Tobacco product preference	Cigars/cigarettes	82	35.3
	E-cigarettes/IQOS	86	37.1
	Midwakh	34	14.7
	Pipes/shisha	30	12.9
Weight management			
Weight management frequency	Daily	20	8.6
	Weekly	85	36.6
	Monthly or less	27	11.6
	Never	100	43.1
Weight changes due to smoking	Yes	51	22.0
	No	95	40.9
	Unsure	86	37.1
Rate satisfaction with weight	Dissatisfied	73	31.5
	Neutral	74	31.9
	Satisfied	85	36.6

As shown in Table 3, only 12.1% of participants reported currently having chronic conditions, and 70.7% were not on any medication. While 79.7% of people who smoke were aware of the risks associated with smoking and obesity, dietary habits were generally poor. Only 21.6% consumed healthy food daily, and 39.7% reported never doing so. Most people who smoke (82.8%) did not follow any diet plans, and 78.8% reported no change in eating habits since beginning smoking. The majority did not perceive any alteration in their weight (70.7%), sense of taste/smell (62.1%), or appetite/food intake (63.4%). Among those who did experience changes in appetite, 66.9% reported a decrease in appetite and food consumption, while 33.1% reported an increase. Regarding the perceived impact on health, 38.8% agreed that smoking has a negative impact, while the majority (61.2%) believed it has no effect. 43.9% of the participants attempted to quit smoking but were unsuccessful, 35.3% of them noticed abdominal obesity while trying to quit, 30.2% reported no abdominal obesity, and 34.5% were unsure about central obesity.

**Table 3:** Knowledge, lifestyle, health behaviors, and the perceived effects of smoking and cessation on weight among participants (*n* = 232).

Variables	Response	Frequency	Percentage (%)
Chronic conditions experienced	Yes	28	12.1
	No	204	87.9
Medication consumption	Yes	68	29.3
	No	164	70.7
Frequency of healthy food consumption	Daily	50	21.6
	A few times a week	73	31.5
	Rarely	17	7.3
	Never	92	39.7
Following any diet	Yes	40	17.2
	No	192	82.8
Aware of smoking and obesity risks	Yes	185	79.7
	No	47	20.3
Eating habits have changed since smoking	Yes	49	21.1
	No	173	78.8
Perceived weight alteration since smoking	My weight increased	43	18.5
	My weight decreased	25	10.8
	No alteration in my weight	164	70.7
Smoking's impact on taste and smell	Yes	88	37.9
	No	144	62.1
Smoking impact on appetite and food intake	Yes	85	36.6
	No	147	63.4
Smoking led to an	Increase in appetite and food consumption	45	33.1
	Decrease in appetite and food consumption	91	66.9
Perceived health impact of smoking	Yes	90	38.8
	No	142	61.2
Smoking cessation attempts	Yes, successfully	60	25.9
	Yes, unsuccessfully	102	43.9
	No, I never tried	70	30.2
Abdominal obesity was noticed during or after cessation attempts	Yes	82	35.3
	No	70	30.2
	Unsure	80	34.5

**Figure 1:**
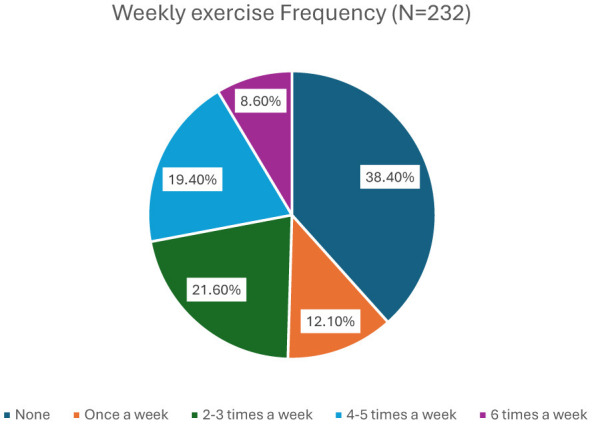
Weekly exercise frequency among participants (*n* = 232). The figure illustrates the proportion of participants who reported exercising at various frequencies per week. The largest percentage (38.4%) reported no exercise, while only 8.6% exercised daily or more.

**Figure 2:**
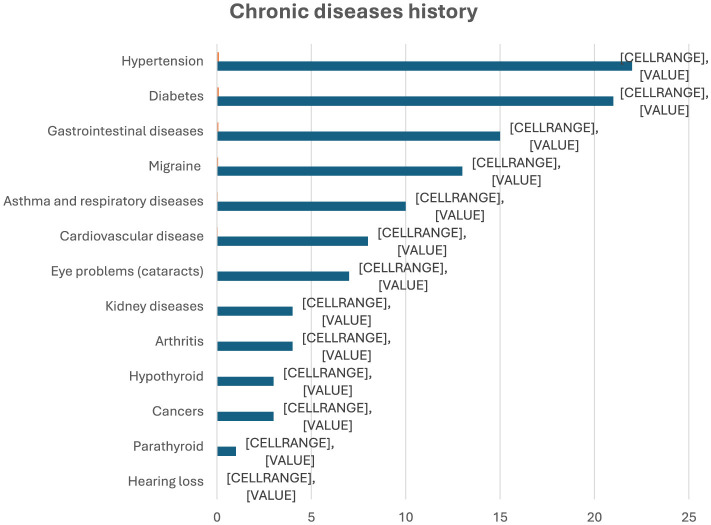
Chronic disease history of the participants. The figure shows the percentage and number of participants who reported specific chronic conditions. The most frequently reported were hypertension (9.5%) and diabetes (9.1%).

As presented in Figure 1, no weekly physical activity was noted among 38.4% of the participants, with many doing none. Prevalence of chronic diseases is summarized in Figure 2; hypertension (9.5%) and diabetes (9.1%) were among the most prevalent. As revealed in Tables 4A, B, prominent relationships between smoking and weight status were reported. Smoking duration was significantly associated with the frequency of weight management (*p* = 0.023). Compared to longer-term people who smoke (>10 years), individuals who smoked for less than 1 year had heterogeneous weight-management behaviors. They were at higher odds of daily weight control (OR = 4.52, 95% CI: 1.27–16.04, *p* = 0.020) and of never engaging in weight management (OR = 5.27, 95% CI: 1.14–24.31, *p* = 0.033). Furthermore, they were 16 times as likely to have reported infrequently managing their weight (monthly or less; OR = 15.82, 95% CI: 1.50–16.39, *p* = 0.021). It is observed that, while the findings reveal infrequent body weight management among new people who smoke, their estimates are accompanied by wide confidence intervals, suggesting unclear precision and a less clear magnitude of influence. Similarly, people with a 1-5-year smoking history showed varied patterns of weight management compared with long-term smokers, they had 9.47 times the odds of not managing weight often (95% CI: 1.61–14.76, *p* = 0.018) and 5.80 times the odds of never managing weight (95% CI: 1.45–23.21, *p* = 0.013). Smoking frequency was also associated with perceived changes in weight (*p* = 0.029). Among daily smokers, the odds of no weight change were 3.2 times higher (95% CI: 1.19–8.71, *p* = 0.02). There was heterogeneity of self-perceived weight outcomes among weekly people who smoke, with increased likelihood of reporting either perceived weight changes (OR = 4.19, 95% CI: 1.10–15.90, *p* = 0.035) or no change (OR = 4.95, 95% CI: 1.33–18.41, *p* = 0.017). BMI categories were significantly associated with smoking duration. Prevalence of normal weight was significantly more for people who smoke with less than 1 year of smoking (66.7%) than for those who smoke for more than 10 years (34.8%; *p* = 0.043). First-year people who smoke had >6 times the odds of achieving a normal weight compared to long-term people who smoke (OR = 6.30, 95% CI: 1.43–29.274, *p* = 0.019), and the confidence interval was even wider, indicating lower precision. Age and sex were also added as variables to all regression models; although the small sample size limits precision, adding these variables did not significantly alter the direction of the significant relationships. To provide a comprehensive picture of the studies conducted, Table 4B has also been updated to include both significant and non-significant connections.

**Table 4A:** Association between smoking habits and weight management habits among participants (*n* = 232).

Smoking variables	Tobacco use frequency			Daily tobacco intake			Smoking duration				Tobacco product preference			
Outcome variables	Daily	Weekly	Monthly	Less than five cigarettes per day	5–10 cigarettes per day	More than 10 cigarettes per day	Less than a year	1–5 years	6–10 years	More than 10 years	Cigars/cigarettes	E-cigarettes/IQOS	Midwakh	Pipes/shisha
Weight management frequency														
Daily	11 (6.4)	5 (16.1)	4 (14.3)	8 (8.6)	6 (8.7)	6 (8.6)	6 (16.7)	4 (7.8)	3 (10.0)	7 (6.1)	9 (11.0)	8 (9.3)	3 (8.8)	0 (0.0)
Weekly	59 (34.1)	13 (41.9)	13 (46.4)	41 (44.1)	19 (27.5)	25 (35.7)	12 (33.3)	22 (43.1)	6 (20.0)	45 (39.1)	28 (34.1)	32 (37.2)	16 (47.1)	9 (30.0)
Monthly or less	19 (11.0)	3 (9.7)	5 (17.8)	15 (16.2)	10 (14.5)	2 (2.9)	7 (19.4)	10 (19.6)	5 (16.7)	5 (4.5)	9 (11.0)	13 (15.1)	1 (2.9)	1 (3.3)
Never	84 (48.6)	10 (32.3)	6 (21.4)	29 (31.2)	34 (49.3)	37 (52.9)	11 (30.6)	15 (29.4)	16 (53.3)	58 (50.4)	36 (43.9)	33 (38.4)	14 (41.2)	17 (56.7)
*p*-Value	0.121			0.053			0.023^*^				0.551			
Weight changes due to smoking														
Yes	34 (19.7)	11 (35.5)	6 (21.4)	20 (21.5)	18 (26.1)	13 (18.6)	10 (27.8)	15 (29.4)	4 (13.3)	22 (19.1)	16 (19.5)	23 (26.7)	7 (20.6)	5 (16.7)
No	76 (43.9)	13 (41.9)	6 (21.4)	37 (39.8)	25 (36.2)	33 (47.1)	8 (22.2)	22 (43.1)	14 (46.7)	51 (44.3)	33 (40.2)	33 (38.4)	19 (55.9)	10 (33.3)
Unsure	63 (36.4)	7 (22.6)	16 (57.1)	36 (38.7)	26 (37.7)	24 (34.3)	18 (50.0)	14 (27.5)	12 (40.0)	42 (36.5)	33 (40.2)	30 (34.9)	8 (23.5)	15 (50.0)
*p*-Values	0.029^*^			0.701			0.126				0.292			
Rate satisfaction with weight														
Dissatisfied	51 (29.5)	13 (41.9)	9 (32.4)	34 (36.6)	21 (30.4)	18 (25.7)	11 (30.6)	18 (35.3)	11 (36.7)	33 (28.7)	19 (23.2)	29 (33.7)	11 (32.4)	14 (46.7)
Neutral	55 (31.8)	6 (19.4)	13 (46.4)	29 (31.2)	19 (27.5)	26 (37.1)	14 (38.9)	15 (29.4)	5 (16.7)	40 (34.8)	30 (36.6)	23 (26.7)	11 (32.4)	10 (33.3)
Satisfied	67 (38.7)	12 (38.7)	6 (21.4)	30 (32.3)	29 (24.0)	26 (37.1)	11 (30.6)	18 (35.3)	14 (46.7)	42 (36.5)	33 (40.2)	34 (39.5)	12 (35.3)	6 (20.0)
*p*-Value	0.153			0.464			0.527				0.534			
BMI categories (calculated from the self-reported height and weight)														
Underweight	8 (4.6)	2 (6.5)	0 (0.0)	5 (5.4)	2 (2.9)	3 (4.3)	2 (5.6)	2 (3.9)	3 (10.0)	3 (2.6)	3 (3.7)	5 (5.8)	2 (5.9)	0 (0.0)
Normal	68 (39.3)	18 (58.1)	13 (46.4)	45 (48.4)	32 (46.4)	22 (31.4)	24 (66.7)	21 (41.3)	14 (46.7)	40 (34.8)	35 (42.7)	40 (46.5)	13 (38.2)	11 (36.7)
Overweight	67 (38.7)	9 (29.0)	11 (39.3)	30 (32.3)	28 (40.6)	29 (41.4)	8 (22.2)	14 (46.7)	8 (26.7)	51 (44.3)	38 (46.3)	24 (27.9)	13 (38.2)	12 (40.0)
Obese	30 (17.3)	2 (6.5)	4 (14.3)	13 (14.0)	7 (10.1)	16 (22.9)	2 (5.6)	8 (15.7)	5 (16.7)	21 (18.3)	6 (7.3)	17 (19.8)	6 (17.6)	7 (23.3)
*p*-Value	0.362			0.199			0.043^*^				0.190			

Bold values and asterisks (^*^) indicate statistically significant results (*p* < 0.05); values are presented as frequency (percentage).

**Table 4B:** Association between smoking habits and weight management frequency among participants (*n* = 232).

Smoking variables	Tobacco use frequency			Daily tobacco intake			Smoking duration				Tobacco product preference			
Outcome variables	Daily	Weekly	Monthly	Less than five cigarettes per day	5–10 cigarettes per day	More than 10 cigarettes per day	Less than a year	1–5 years	6–10 years	More than 10 years	Cigars/cigarettes	E-cigarettes/IQOS	Midwakh	Pipes/shisha
Weight management frequency														
Daily	0.024^*^ OR = 0.196 95% CI; 0.048, 0.807	NS	NS	NS	NS	NS	0.020^*^ OR = 4.519 95% CI; 1.273, 16.040	NS	NS	NS	NS	NS	NS	NS
Weekly	0.031^*^ OR = 0.324 95% CI; 0.117, 0.902	NS	NS	0.037^*^ OR =2.092 95% CI; 1.044, 4.194	NS	NS	NS	NS	NS	NS	NS	NS	NS	NS
Monthly or less	NS	NS	NS	NS	NS	NS	0.021^*^ OR = 15.815 95% CI; 1.504, 16.389	0.018^*^ OR = 9.467 95% CI; 1.608, 14.762	NS	NS	NS	NS	NS	NS
Never	NS	NS	NS	0.033^*^ OR = 5.741 95% CI; 1.151, 28.649	NS	NS	0.033^*^ OR = 5.273 95% CI; 1.143, 24.313	0.013^*^ OR = 5.80 95% CI; 1.449, 23.209	NS	NS	NS	NS	NS	NS
*p*-Value	0.113			0.015^*^			0.015^*^				0.264			
Weight changes due to smoking														
Yes	NS	0.035^*^ OR = 4.190 95% CI; 1.104, 15.901	NS	NS	NS	NS	NS	NS	NS	NS	NS	NS	NS	NS
No	0.021^*^ OR = 3.217 95% CI; 1.188, 8.709	0.017^*^ OR = 4.952 95% CI; 1.332, 18.414	NS	NS	NS	NS	0.034^*^ OR = 0.366 95% CI; 0.25, 0.535	NS	NS	NS	NS	NS	0.030^*^ OR = 3.562 95% CI; 1.128, 11.252	NS
Unsure	NS	NS	NS	NS	NS	NS	NS	NS	NS	NS	NS	NS	NS	NS
*p*-Value	0.029^*^			0.705			0.104				0.297			
Rate satisfaction with weight														
Dissatisfied	NS	NS	NS	NS	NS	NS	NS	NS	NS	NS	0.014^*^ OR = 0.247 95% CI; 0.081, 0.749	NS	NS	NS
Neutral	NS	NS	NS	NS	NS	NS	NS	NS	NS	NS	NS	NS	NS	NS
Satisfied	NS	NS	NS	NS	NS	NS	NS	NS	NS	NS	NS	NS	NS	NS
*p*-Value	0.649			0.464			0.482				0.200			
Calculated BMI categories														
Underweight	NS	NS	NS	NS	NS	NS	NS	NS	NS	NS	NS	NS	NS	NS
Normal	NS	NS	NS	0.042^*^ OR = 2.517 95% CI; 1.032, 6.142	0.024^*^ OR = 3.325 95% CI; 1.174, 9.415	NS	0.019^*^ OR = 6.30 95% CI; 1.356, 29.274	NS	NS	NS	0.045^*^ OR = 3.712 95% CI; 1.028, 13.401	NS	NS	NS
Overweight	NS	NS	NS	NS	NS	NS	NS	NS	NS	NS	0.044^*^ OR = 3.694 95% CI; 1.039, 13.142	NS	NS	NS
Obese	NS	NS	NS	NS	NS	NS	NS	NS	NS	NS	NS	NS	NS	NS
*p*-Value	0.233			0.193			0.042^*^				0.109			

NS, not significant; OR, odds ratio; CI, confidence interval.

Bold values and asterisks (^*^) indicate statistically significant results (*p* < 0.05); values are presented as frequency (percentage).

Table presents statistically significant associations only; non-significant results are available upon request.

## 4 Discussion

This study offers key insights into the complexities of the relationship between smoking and weight management among adult people who smoke in the UAE. Previous studies have reported several associations between smoking and weight outcomes, but exploring those associations in terms of self-regulation and monitoring behaviors within the context of high levels of overweight and obesity prevalence in the UAE yields additional insights. In this study, the majority of the people who smoked were male, demonstrating that males continue to smoke more often than females. This could be due to cultural norms, sex roles, and targeted marketing that made tobacco use socially more acceptable (40).

The results of this study can be interpreted considering the Theory of Planned Behavior. Smoking frequency, smoking duration, and cessation attempts can be considered behavioral constructs, whereas weight management practices and weight change can be considered attitude constructs. The results showed that weight management practices vary across smoking durations and frequencies, which can be considered a construct of perceived behavioral control and behavioral orientation among smokers. This shows that Theory of Planned Behavior (TPB) can be considered a valid framework for interpreting the results.

Similar sex-based disparities were observed in studies from Saudi Arabia and the UAE (2, 26), indicating larger regional trends. The mean BMI calculated from the anthropometric measurements of the study participants was 26.0259 ± 5.55 kg/m^2^. According to the WHO classification, a BMI of 18.5–24.9 is considered normal weight (41). However, with a mean BMI above 25, the study participants, on average, fall into the overweight category, thereby increasing their risk. Regarding physical activity levels, the data showed that a notable portion of people who smoke reported sedentary or low levels of physical activity. This suggests that many people who smoke may not engage in sufficient exercise or movement, which is a well-established link between physical inactivity and various health risks, including cardiovascular diseases, diabetes, and obesity (42).

This means many participants might not get enough exercise or movement, which is a concern because it is well-established that physical inactivity is linked to a multitude of health risks (e.g., cardiovascular diseases, diabetes, obesity) (42). Self-reported frequency of physical activity may not properly capture the variation in participants' activity levels. Furthermore, variation in dietary patterns (due to the representation of nationally diverse populations) may contribute to differences in weight outcomes, alongside differences in smoking habits.

About 43.1% of participants reported never monitoring their weight, despite a high percentage (79.7%) being aware of the risks of smoking and obesity. Additionally, this risk awareness did not translate into healthier habits, as only 21.6% reported daily consumption of healthy foods, and the vast majority (78.8%) hadn't changed their eating patterns since they began smoking. The lack of a gradient between knowledge and action suggests that awareness does not automatically lead to sustained self-regulation. From the TPB viewpoint, once people recognize health consequences, they may have weak intentions to change due to lower perceived behavioral control and practical and motivational barriers that obstruct translating that risk awareness into routine behaviors. Overall, among participants in our study, 36.6% reported that smoking influences food intake, including two-thirds (66.9%) who reported their appetite and/or food consumption decreased, and one-third (33.1%) who reported an increase. This dichotomy may be attributed in part to variations in individual response and exposure time, as nicotine has previously been shown to work in the short-term at modifying appetite management, but this effect is expected to diminish with later use (43). Consistent with that, daily people who smoke on a habitual basis were found to be less overweight than those woh don't smoke which suggests that earlier appetite suppressants of nicotine might not be maintained in long-term smokers. Although such mechanisms should not be considered direct explanatory features underlying the observed associations, they are nonetheless worth considering as possibilities. Furthermore, although psychological outcomes of stress and depressive symptoms were not included in the analyses used in the present work, and while they do not appear to determine physical health, these can contribute to eating behaviors and smoking urges, which may account for the differences in weight outcomes. The tobacco products of choice varied from participant preferences, with electronic cigarettes/IQOS as the most commonly used tobacco products by participants, with Midwakh and shisha as the second choices. Additionally, 35.3% reported abdominal obesity during cessation attempts, and many were unsuccessful. This highlights the difficulty of quitting smoking and its effect on the weight status of some individuals, and may prevent smoking cessation, and may even be a barrier for an individual to quit despite being aware of the risk for health and perceived low control over weight (34). This may be covered by practical barriers to behavioral change, under TPB. The result is in line with previous findings indicating that weight gain upon cessation may occur when caloric intake increases and resting energy expenditure declines after nicotine withdrawal (43).

Smoking duration and frequency were found to shape weight-management behaviors, perceived weight change, and BMI in distinct ways. People who have been smoking for one year had more variable monitoring habits compared to long-term people who smoke (>10 years). Some engaged in frequent monitoring, while others did not. A similar pattern was observed among those with 1–5 years of history, who had inconsistent monitoring frequencies. These patterns may reflect differences in attitude and behaviors toward weight control among early people who smoke. On the other hand, long-term people who smoke may have more established weight-related habits, active or inactive, that are not influenced by smoking behavior. Smoking duration also emerged as a predictor of BMI category. In our study, first-year people who smoke were more likely to be in the normal BMI category than the other groups (OR = 6.30, *p* = 0.019). However, population-based evidence suggests that smoking–obesity patterns differ across subgroups and it depends on the exposure definitions and therefore should not be interpreted as a uniform predictor of BMI category (20). No significant associations were found between tobacco product type (cigarettes vs. e-cigarettes/IQOS vs. Midwakh/shisha) and weight management frequency, reported weight changes, weight-satisfaction ratings, or BMI categories. This finding is contrary to a study in Qatar, which found higher BMI and fat mass among waterpipe users (1). Likewise, among university students in Dubai (UAE) and the West Bank, waterpipe smoking showed a significant positive association with overweight/obesity, and some reported smoking waterpipe as a means to reduce weight, highlighting that beliefs about weight control can shape smoking status (2). Additionally, smoking frequency was linked to self-perceived weight change among people who smoke weekly who reported both higher perceived weight change and no change at all. In contrast, there was no statistically significant effect in most models for daily tobacco intake (*p* > 0.05). This observation is also consistent with the general literature, which reports mixed results depending on definitions (e.g., objective BMI vs. perceived weight change; general population vs. people who smoke only; product type) (1, 2, 20). As a whole, the results suggest that smoking behavior was associated with differences in weight control habits and perceived weight outcomes among the sample in our study, with greater variability observed during early smoking stages. To our knowledge, this study is among the first to examine the link between smoking behaviors and weight control in the UAE, which has unique patterns in this regard.

## 5 Limitations

The sample size limitation of this study, due to time and resource constraints, may limit the generalizability of the findings. As the topic is sensitive, it became difficult to recruit participants, as some people who smoke were reluctant to participate despite assurances of confidentiality and privacy provided in the consent form. A cross-sectional study design was also a limitation, as data were collected at a single point in time, and causation could not be established between smoking behaviors and weight management. Moreover, the survey design was conducted online, and individuals without internet access or digital skills might have been excluded from the sample. As snowball sampling is a non-probability sampling method, it has the potential to over-represent certain demographic characteristics, such as younger respondents, in communities like the Emirates of Dubai, Abu Dhabi, and Sharjah. By taking this approach, generalizability to the entire population is limited. Self-reports of smoking behavior, weight management practices, and weight change perception were at risk of recall and social-desirability bias. Future studies must employ longitudinal designs (e.g., prospective cohort studies) to examine smoking initiation, changes in smoking intensity, quitting, and weight-related alterations over time. To improve theoretical integration, future research should include standardized TPB measures that evaluate attitudes, subjective norms, perceived behavioral control, and behavioral intention.

## 6 Conclusion and recommendations

The current study demonstrated that smoking habits correlate with BMI as well as patterns of weight management. Reduced periods of smoking were associated with an inconsistent frequency of weight management and increased odds of living within normal BMI groups. Frequency of smoking was linked to perceived weight change, with higher odds of weekly people who smoke reporting both weight change and no weight change. These findings highlight the importance of developing interventions based on smoking frequency and history, as well as the effects of smoking frequency and duration to prevent smoking, which may be especially helpful in addressing weight issues related to smoking risk behaviors and smoking cessation attempts. Adopting weight management recommendations and interventions, including dietitian linkage, brief exercise suggestions, and follow-up on weight changes, could help overcome barriers that dissuade cessation or hinder consistent behavior change. The observed use of e-cigarettes and heated tobacco products highlights the ongoing relevance of regulatory mechanisms, including marketing restrictions, flavor bans, health and weight warnings, strict age checks, and taxation to deter tobacco use. Although regulatory measures (such as taxes, flavor bans, and marketing restrictions) remain crucial to broader tobacco control initiatives, however, tobacco control efforts that take weight management behaviors into account might help enable broader public health strategies that lead to better long-term health outcomes, as well as smoking cessation.

## Data Availability

The original contributions presented in the study are included in the article/supplementary material, further inquiries can be directed to the corresponding author.
